# From speculation to regulation: an evidence-based analysis of the EU reclassification of non-invasive brain stimulation devices

**DOI:** 10.1093/jlb/lsag021

**Published:** 2026-08-26

**Authors:** Roman Rethwilm, Karsten Litschel, Desmond Agboada, Wolfgang Mack, Thomas Weyh, Wolfgang Seiberl

**Affiliations:** Institute of Sport Science, University of the Bundeswehr Munich, Neubiberg 85579, Germany; Institute of Energy Engineering, University of the Bundeswehr Munich, Neubiberg 85579, Germany; Institute of Psychology, University of the Bundeswehr Munich, Neubiberg 85579, Germany; Institute of Psychology, University of the Bundeswehr Munich, Neubiberg 85579, Germany; Institute of Energy Engineering, University of the Bundeswehr Munich, Neubiberg 85579, Germany; Institute of Sport Science, University of the Bundeswehr Munich, Neubiberg 85579, Germany

**Keywords:** evidence-based policymaking, medical device regulation (MDR), neurotechnology governance, non-invasive brain stimulation (NIBS), regulatory impact, risk classification

## Abstract

In December 2022, the European Commission reclassified non-invasive brain stimulation (NIBS) devices without a medical purpose into the highest risk category (Class III) under Regulation (EU) 2022/2347, referring to ‘available scientific evidence’ on potential neurological risks. This article examines the evidentiary basis and regulatory implications of that decision. A systematic analysis of the five references submitted by six Member States showed that the list of side effects incorporated into the legal text was largely drawn from a single ethics-oriented publication not intended to assess clinical safety. None of the cited sources include empirical investigations of device safety or systematic reviews of adverse events. In contrast, an extensive body of clinical research comprising hundreds of randomized controlled trials and meta-analyses provides a detailed empirical account of the safety profile of NIBS techniques. The resulting framework places non-medical NIBS devices in the same category as highest-risk medical technologies, with observable effects manifesting. Furthermore, the codification of potential side effects in legally binding text may influence institutional and public perceptions, shaping research governance and ethics reviews. The paper concludes by recommending a regulatory approach that reflects user competence, evaluates risks according to probability, and integrates expert consultation to ensure consistency with evidence-based lawmaking.

## I. INTRODUCTION

Science is necessary for understanding the natural and social world, relying on systematic observation, experimentation, and critical analysis to generate reliable knowledge. Evidence-based insights help to distinguish facts from assumptions, ensuring that decisions are grounded in reality rather than speculation. This approach is especially crucial when it comes to the development of laws and regulations. Policies based on rigorous scientific evidence help to create legal frameworks that are not overly strict and promote innovation and technological advancements while protecting society by minimizing risks. Without a solid foundation in evidence-based knowledge, regulations may be ineffective, misguided, or even harmful.

With the implementation of the European Union (EU) Medical Device Regulation (MDR) (EU 2017/745) in 2021, a significant change to the previous Medical Device Directive (MDD) (93/42/EEC) was the integration of device groups without medical purpose which must also now comply with the MDR. These types of devices previously only had to comply with general product safety requirements and were not subjected to the comprehensive regulations for medical devices, such as risk management, clinical evaluation, and post-market surveillance. The integration of device groups without medical purpose was introduced to prevent companies from having an easy pathway to market devices with a non-medical intended use, such as exercise, well-being, or cognitive enhancement purposes. Since these devices typically were technically identical to a medical device, they come with the same inherent risks. Closing this regulatory gap resulted in the list of devices without medical purposes (Annex XVI, MDR), which now fall within the scope of the MDR.

In addition, the Commission further published two more Implementation Regulations regarding devices within Annex XVI in December 2022:

1) Implementation Regulation (EU) 2022/2346, laying down common specifications for risk management and general safety and performance requirements.
2) Implementation Regulation (EU) 2022/2347, reclassifying devices for non-invasive brain stimulation (NIBS) into the highest risk category class III.

Devices for NIBS are often used for medical purposes to treat or diagnose psychiatric or neurological conditions by modulating neural activity,^1^ thereby clearly identifying as medical devices. However, NIBS devices are also employed for non-medical purposes, such as for cognitive enhancement,^2^ performance optimization,^3^ mood and well-being improvement,^4^ sleep regulation,^5^ and creativity stimulation.^6^ Many of these applications align with the growing interest in consumer neurotechnology, where NIBS devices are advertised for everyday use in enhancing cognitive function, productivity, and overall well-being. Not only must all of these devices comply with the MDR due to Annex XVI, but they are also classified in the highest risk category, which entails stricter regulatory requirements. Among other obligations, and with very few exceptions, clinical investigations are mandatory for Class III devices, as stipulated in MDR Article 61(4). Given the high costs of clinical investigations and the additional regulatory burdens, NIBS devices without medical purpose are at risk of being withdrawn or never certified for the EU market—a trend already evident in high-risk devices for pediatric cardiology and rare diseases, where manufacturers have indicated that the high costs relative to small market volumes make recertification unviable.^7^

Basic scientific research is another use case for NIBS devices, where the underlying neurophysiological mechanisms and potential benefits and risks are broadly investigated. Previously, under the Medical Device Directive, NIBS devices could be marketed with non-medical purpose, allowing scientists to investigate neurophysiological mechanisms without the need for full medical certification. For the same reasons mentioned above, especially in Europe, researchers in the field of brain stimulation voice concern that their ability to explore new applications of NIBS could be severely restricted, potentially stalling scientific progress in neurophysiology and translational medicine,^8^ which could ultimately prompt European researchers to relocate their work abroad.

Furthermore, much critique was voiced by the scientific community that experts in the field of NIBS were not involved in the law-making process and that the reclassification is built on ‘gravely flawed’ evidence.^9^ This directly contradicts the EU Commission’s commitment to evidence-based policy- and law-making, including stakeholders’ consultations.^10^ The lack of consultation with field experts raises serious concerns about the scientific integrity of the decision-making process, suggesting that EU-wide binding regulatory changes were made without adequately considering established research and clinical expertise. As a result, the reclassification appears to be driven more by precautionary principles rather than a balanced assessment of risks and benefits, potentially leading to disproportionate regulatory barriers that were not properly scrutinized.

With this paper we aim to critically examine the scientific evidence cited to justify the reclassification of non-medical NIBS devices under the MDR. In the following we highlight fundamental shortcomings in the assessment of risks and the resulting disproportionate regulatory consequences. Building on current practices in neuroscience, clinical application, and technical perspectives, the current analysis critically examines the shortcomings of Implementing Regulations (EU) 2022/2346 and (EU) 2022/2347. We further discuss the wider legal and practical implications of this regulatory approach, including its potential to restrict scientific research and innovation in Europe. Finally, we outline elements of an alternative, evidence-based regulatory framework designed to ensure safety while avoiding unnecessary barriers to research, accessibility, and technological development.

## I‌I. BACKGROUND AND HISTORY OF THE RECLASSIFICATION

In the EU, the MDR establishes a risk-based classification system for medical devices, ranging from Class I (low risk) to Class III (high risk), based on their intended use and potential risks. According to Article 51(3)(b), devices may be reclassified by the European Commission in response to new scientific evidence or information obtained through market surveillance.

In July 2022, six Member States (Belgium, Netherlands, Portugal, France, Spain, and Luxembourg) submitted a formal request to the European Commission to reclassify brain stimulation devices without a medical purpose from Class I to Class III. This request was supported by a literature research, indicating that devices applying electrical currents or electromagnetic fields for transcranial stimulation (ie NIBS) pose potential safety risks. The Member States’ request was later published on the EU Transparency Portal and included a list of identified side effects, based on five literature sources related to NIBS devices.

The Commission, following consultation with the Medical Devices Coordination Group (MDCG), opened a public feedback period from August to September 2022 and subsequently adopted the reclassification through Commission Implementing Regulation (EU) 2022/2347.

However, an industry stakeholder filed a complaint with the European Ombudsman in March 2023, arguing that the Commission had not followed a sufficiently transparent, evidence-based, and participatory process in reaching its decision.^11^ In response, the Ombudsman launched an inquiry and recommended that the Commission verifies that the scientific evidence provided by the Member States is comprehensive and representative of the most recent scientific evidence concerning the risks of brain stimulation (Case 1157/2023/VB – www.ombudsman.europa.eu).

In March 2024, the Scientific Committee on Health, Environmental and Emerging Risks (SCHEER) was tasked with assessing the most recent scientific evidence, but only including studies published after April 2017. The resulting SCHEER opinion: health hazards and risks associated with the use of brain stimulators for non-medical purpose as indicated in group 6 of Annex XVI to Regulation (EU) 2017/745,^12^ now available as a preliminary version, provides a comprehensive and systematic evaluation of the current evidence base on NIBS, including an assessment of health hazards and associated risks. The SCHEER report substantially expands the evidentiary foundation compared with the limited sources originally used to justify the reclassification and its findings are broadly consistent with the established scientific consensus within the field.

### I‌I.A. MDR Definition of NIBS Devices

To contextualize the regulatory discussion, it is first necessary to clarify what technologies are encompassed by the definition of the MDR. NIBS devices that fall under the definition of the MDR are only those that ‘apply electrical currents or magnetic or electromagnetic fields that penetrate the cranium to modify neuronal activity in the brain’ (Annex XVI(6), MDR). Devices utilizing electrical currents include transcranial electrical stimulation (tES) devices, which can be further categorized into transcranial direct current stimulation (tDCS), transcranial alternating current stimulation (tACS), and transcranial random noise stimulation (tRNS).^13^ Devices utilizing electromagnetic fields include transcranial magnetic stimulation (TMS), which can be facilitated for a variety of different applications, including research.^14^ Cranial electrotherapy stimulation (CES) occupies a regulatory and neurophysiological gray zone between peripheral and central nervous systems neuromodulation. This technique applies weak alternating or pulsed electrical currents—typically via electrodes placed on the earlobes, temples, or mastoid areas—to stimulate cranial nerves such as branches of the vagus or trigeminal nerves.^15^ Through these afferent pathways, CES indirectly influences neuronal activity, modulating arousal, mood, or sleep-related circuits via projections to brainstem and cortical regions. Although a small fraction of current may enter the cranium during CES, the resulting field strengths are far below the threshold required to induce direct neuronal modulation. As CES primarily targets cranial nerves rather than cortical neurons, any changes in brain activity occur indirectly through afferent pathways. Consequently, CES should not be considered within the scope of NIBS devices as defined under Annex XVI (6) of the MDR.

## I‌II. THE EVIDENCE FOR THE RECLASSIFICATION

The six Member States submitted five references that were intended to substantiate potential safety risks associated with NIBS devices. These references included a mix of academic, policy, and ethical sources rather than empirical safety studies: two publications by Maslen et al. on the ethical and regulatory aspects of cognitive enhancement technologies,^16^ a policy report by the Nuffield Council on Bioethics,^17^ one neurophysiological study by Monte-Silva et al.,^18^ and a public perception survey by Wagner et al.^19^ According to Antal et al. these sources formed the entire evidentiary basis upon which the reclassification request was justified and later reflected in Implementing Regulation (EU) 2022/2347.^20^ Moreover, their analysis revealed that the five references were insufficient, as none of the sources reviewed NIBS safety nor directly provided clinical safety data.^21^ While Antal et al. already outlined major flaws, the current paper takes an even deeper look into the evidence provided that shaped this EU law.

In our analysis, we systematically examined the occurrence, context, and sequence of side effects across the five references provided by the Member States to justify the reclassification of NIBS devices. First, we analyzed if and how many times each side effect (eg atypical brain development) was mentioned in each source in a context of side effects or safety and to which NIBS device it was attributed to. The results are displayed in Table 1.

**Table 1:** Side effects referenced by Regulation (EU) 2022/2347, their counts of occurrence (parentheses) in the context of side effects and safety of a device (Device type) in the five references provided by the six EU Member States. Superscript numbers indicate the references (1-5). Abbreviations: transcranial direct current stimulation (tDCS), neurofeedback (training), deep brain stimulation (DBS), cranial electrotherapy stimulation (CES), transcranial magnetic stimulation (TMS).

NIBS side effects listed in Regulation (EU) 2022/2347	Device type	Maslen et al., 2014a ^1^	Maslen et al., 2014b Mind Machines ^2^	Monte-Silva et al., 2018 ^3^	Nuffield Council on Bioethics, 2013 ^4^	Wagner et al., 2018 ^ 5^
		Occurrence	Occurrence	Occurrence	Occurrence	Occurrence
**Atypical brain development**	**tDCS** ^**1**^ ^**,**^ ^**2**^	Yes (1)	Yes (1)	No (0)	No (0)	No (0)
**Abnormal brain activity**	**tDCS** ^**1**^ ^**,**^ ^**2**^	Yes (1)	Yes (1)	No (0)	No (0)	No (0)
**Increased metabolic consumption**	**tDCS** ^**1**^ ^**,**^ ^**2**^	Yes (1)	Yes (1)	No (0)	No (0)	No (0)
**Fatigue**	**Neurofeedback** ^**1**^ ^**,**^ ^**2**^	Yes (1)	Yes (1)	No (0)	No (0)	No (0)
**Anxiety**	**Neurofeedback** ^**1**^ ^**,**^ ^**2**^, **DBS**^**4**^	Yes (2)	Yes (1)	No (0)	Yes (2)	No (0)
**Irritability**	**Neurofeedback** ^**1**^ ^**,**^ ^**2**^	Yes (1)	Yes (1)	No (0)	No (0)	No (0)
**Headaches**	**CES** ^**1**^ ^**,**^ ^**2**^, **Neurofeedback**^**1**^^**,**^^**2**^	Yes (2)	Yes (3)	No (0)	No (0)	No (0)
**Muscle twitches**	**Neurofeedback** ^**1**^, **TMS**^**4**^	Yes (1)	No (0)	No (0)	Yes (1)	No (0)
**Tics**	**Neurofeedback** ^**1**^	Yes (1)	No (0)	No (0)	No (0)	No (0)
**Seizures**	_**TMS**_ **^**2**^ ^,^ ^**4**^**	No (0)	Yes (3)	No (0)	Yes (2)	No (0)
**Vertigo**	**CES** ^**2**^	No (0)	Yes (2)	No (0)	No (0)	No (0)
**Skin irritation**	**CES** ^**2**^	No (0)	Yes (1)	No (0)	No (0)	No (0)

[1] Maslen, Hannah, Douglas, Thomas, Cohen Kadosh, Roi, Levy, Neil, and Savulescu, Julian. “The regulation of cognitive enhancement devices: extending the medical model.” Journal of Law and the Biosciences 1, no. 1 (2014): 68–93.

[2] Hannah Maslen, Thomas Douglas, Roi Cohen Kadosh, Neil Levy, Julian Savulescu. “Mind Machines: the regulation of cognitive enhancement devices.” The Oxford Martin School 2014.

[3] Monte-Silva, Katia, Kuo, Min-Fang, Hessenthaler, Silvia, Fresnoza, Shane, Liebetanz, David, Paulus, Walter, and Nitsche, Michael A. “Induction of Late LTP-Like Plasticity in the Human Motor Cortex by Repeated Non-Invasive Brain Stimulation.” Brain stimulation 6, no. 3 (2013): 424–432.

[4] Novel neurotechnologies: Intervening in the brain. London: Nuffield Council on Bioethics, 2013.

[5] Wagner, Katy, Maslen, Hannah, Oakley, Justin, and Savulescu, Julian. “Would you be willing to zap your child's brain? Public perspectives on parental responsibilities and the ethics of enhancing children with transcranial direct current stimulation.” AJOB empirical bioethics 9, no. 1 (2018): 29–38.

All of the side effects listed in Regulation (EU) 2022/2347 can be reproduced when combining the two Maslen et al. sources (see Table 1). These texts should effectively be treated as a single source, since *Mind Machines* is a companion paper derived from the original academic article. Strikingly, the sequence of side effects in the EU legal text is almost identical to that presented in the original paper,^22^ with the sole exception that the latter omits the last three items (seizure, vertigo, and skin irritation). These three side effects, however, appear in the companion paper.^23^ As shown in Table 1, the entire list and ordering of side effects in the regulation can thus be traced directly to the two Maslen et al. sources. This strongly suggests that the two respective papers by Maslen et al. served as the primary conceptual source for the legal text, while the remaining references appear to have played a supplementary role.

The contextual analysis of side effects in terms of device attribution reveals another flaw in the evidence base of the legal text: only five of the 12 side effects were mentioned in relation to NIBS devices in the five references provided by the six EU Member States (Table 1). The majority of side effects are mentioned in a different context, such as neurofeedback training, deep brain stimulation or CES. This finding suggests a lack of rigor in the evidence review and decision-making process, indicating that neither the scientific assessment nor the regulatory scrutiny met the standards expected for evidence-based policymaking, despite the Regulation’s claim that the MDCG had been consulted in the process (Recital 9, (EU) 2022/2347).

To put it in plain terms, the side effects listed in a legally binding document are not only almost entirely derived from a single source, which is arguably inappropriate to assess the safety of the devices in question, but they were also attributed to NIBS devices without properly verifying which specific devices they pertain to in the primary source of evidence.

While some side effects are well-documented and widely accepted, others appear to be purely hypothetical within the context of the reclassification justification. Among hypothetical side effects, atypical brain development and abnormal brain activity stand out as the most severe and concerning claims. Yet, they do not align with the known side effects documented in current NIBS safety literature, which we will examine in more detail below.

Before analyzing the cited evidence for the two hypothetical side effects (atypical brain development and abnormal brain activity), it is important to emphasize that we do not intend to discredit the work of Maslen et al. in any way. Their paper provides valuable insights into legal and ethical considerations, and it is clear that their cited literature was not intended to serve as a comprehensive safety review but rather to support their broader regulatory arguments.

For **atypical brain development** Maslen et al. cite only one paper by Spencer-Smith & Anderson which describes healthy and abnormal development of the prefrontal cortex.^24^ While it provides insights into brain maturation and lesion-induced abnormalities, it does not study or demonstrate that NIBS devices lead to atypical brain development. Therefore, this citation only serves to illustrate how brain lesions can affect the maturing brain, but it does not establish any evidence that NIBS devices cause brain lesions or atypical brain development.

Maslen et al. referenced **abnormal patterns of brain activity** by citing and closely mirroring the wording of another neuroethics paper, which speculated that such effects could pose a potential risk but provided no empirical data or supporting references.^25^ The statement therefore remains speculative and is not substantiated by scientific evidence.

Recital (7) of Regulation (EU) 2022/2347 argues for the reclassification that neuromodulative effects may have long-lasting and potentially irreversible consequences. The discussion of long-term effects is also present in Maslen et al., which reference a study that quantified long-term neuromodulative effects using two behavioral tasks done before and after a training as well as six months post-intervention.^26^ However, a significant limitation of this study is that for the six-month follow-up only the intervention group was invited, without a sham control, making it challenging to isolate the specific effects of brain stimulation from other potential influences. Notably, and probably even more important in the context of our current paper, no adverse effects were reported in this study.

The only other reference cited in support of the reclassification, where some of the side effects were mentioned, is the 2013 policy paper on novel neurotechnologies by the Nuffield Council on Bioethics.^27^ The foreword states: *‘It is not the aim of this report to provide a detailed clinical assessment of the safety and efficacy of these novel technologies; instead we aim to provide a reflective assessment of the ethical and social issues that are raised by their development and use.’.*^28^ Founding their statements on established safety literature and reviews (eg Rossi et al.),^29^ they conclude about the risks and safety of NIBS devices: *‘[…] there is little danger from the application of TMS or DC/AC currents per se.’.* Furthermore, *‘[…] the major theoretical risk with using TMS is of inducing an epileptic seizure. However, current guidelines and exclusion criteria make this risk extremely small.’.*  ^30^ It is speculative to assume that this 267-page source was too extensive for member state officials and lawmakers to read in full. However, given that its conclusions directly contradict the arguments presented for the reclassification, this remains a plausible explanation.

The inclusion of the well-regarded Monte-Silva et al. paper in the references can only be speculated upon. A plausible explanation is that it demonstrates the potential for neuromodulative effects. However, it is important to note that the study did not report any adverse effects, as explicitly stated by the authors.^31^

Lastly, the paper by Wagner et al. was cited in support of the reclassification. ^32^ While it may capture aspects of general lay opinion regarding the application of neuroenhancement in children, its title *‘Would you be willing to zap your child’s brain?’* and some of the questions appear to be quite suggestive and therefore limit its value as an objective source of evidence.^33^ Moreover, the paper does not report any of the side effects listed in the regulation, and, as others have noted, it does not provide empirical data relevant to safety.^34^ Its role in supporting the reclassification thus appears to be of limited relevance.

Regulation (EU) 2022/2347 applies to devices regulated under the MDR, which imposes strict requirements on the clinical evaluation of medical devices, including the quality and robustness of supporting scientific literature. These requirements are further detailed in the guidance document MEDDEV 2.7/1 Revision 4, which provides a framework for assessing clinical data. Annex 6 (A6) of this guidance highlights examples of a lack of scientific validity, explicitly categorizing ‘hypothesis papers and unsubstantiated opinions’ as insufficient evidence for clinical evaluation. Especially the aforementioned hypothetical side effects (*atypical brain development* and *abnormal patterns of brain activity*) arguably fall within this category of unsuitable evidence. Rigorous assessment and quality checks should safeguard not only the validity of scientific and clinical data but also the integrity of law-making, ensuring that policies are grounded in solid evidence rather than speculation.

## IV. EMPIRICAL EVIDENCE OF THE SAFETY OF NIBS DEVICES

NIBS is a well-established technique and has been applied for decades. The first TMS device meant clinical applications was developed in 1985.^35^ Later, in the early 2000s, modern tDCS resurged,^36^ particularly after the influential study by Nitsche and Paulus, which demonstrated that weak transcranial direct current stimulation can change excitability in the human motor cortex.^37^ During this time span, extensive research has been conducted to investigate the safety and efficacy of NIBS devices. Numerous studies have examined their neurophysiological effects,^38^ therapeutic and diagnostic potential,^39^ and safety profiles across various applications.^40^ In an initial systematic literature search on PubMed alone, we identified 355 (n = 210 TMS; n = 145 tES) clinical trials and randomized controlled trials (RCT) when searching for the different NIBS devices in connection with safety (see Supplemental data). This substantial body of empirical research data encompasses various stimulation protocols for a wide range of pathologies, forming the foundation for numerous systematic reviews and meta-analyses addressing the safety of NIBS devices. This body of empirical studies outweigh the five references cited in support of the reclassification—references that do not meet the minimum criteria for scientific evidence required by the guidelines (MEDDEV 2.7/1 Revision 4) used to evaluate the safety of the very group of devices now subjected to the reclassification.

### IV.A. SCHEER Opinion: Report on Current Evidence

The SCHEER opinion comprises a comprehensive evaluation of the most recent literature on NIBS, focusing on studies published after April 2017.^41^ In total, 907 relevant sources were evaluated, including medical and non-medical research articles, experimental studies, systematic reviews, and meta-analyses. This assessment represents the most extensive and methodologically structured synthesis of current evidence on the safety of TMS and tES.

Across the reviewed literature, the SCHEER report characterizes both TMS and tES as generally safe when applied within established protocols and in accordance with appropriate screening and operational procedures.^42^ Reported adverse effects are predominantly mild and transient, including headache, local discomfort, or skin irritation. Serious adverse events, such as seizures in the context of TMS, are described as rare.

Importantly, the SCHEER opinion does not identify evidence for persistent or progressive adverse effects under standard conditions of use.^43^ Instead, the overall risk profile is described as dependent on stimulation parameters, individual susceptibility, and conditions of application, including the presence of appropriate screening, trained personnel, and professional supervision. Therefore, reflecting a conditional, context-dependent risk profile, rather than an intrinsically high-risk technology.

### IV.B. Evidence vs. Risks

While both *clinical evaluation* and *risk management* are essential components of medical device regulation, they differ fundamentally in their approach to evidence and potential risks. Clinical evaluation relies primarily on empirical data—such as clinical trials, published literature, and post-market surveillance—to demonstrate the safety and performance of a device under intended conditions of use. In contrast, *risk management* takes a broader and more proactive stance, aiming to identify, and evaluate all reasonably foreseeable hazards, including those without prior clinical observation but which are scientifically plausible. Sound regulation demands an evidence-based and risk-based approach that accommodates future misuse scenarios while remaining proportionate to the actual evidence of harm.

The reclassification of non-medical NIBS devices into Class III is framed around the precautionary principle and the potential for serious neurological risks. However, two critical issues arise. First, by explicitly stating that the decision is based on ‘available scientific evidence’ [(EU) 2022/2347, Recital 7], the regulation implies the presence of a sufficient and valid evidence base. However, as illustrated above and criticized by others,^44^ the cited literature for the reclassification does not meet the evidentiary standards required to support such a claim. Second, even under a precautionary risk management approach—where the goal is to identify and evaluate all reasonably foreseeable risks, including those not yet clinically observed—the provided evidence fails to establish scientific plausibility. At a minimum, such plausibility would require mechanistic support from in vitro studies, preclinical models, or analogies with similar interventions. None of the referenced sources offer such substantiation. As a result, some of the most severe hypothetical risks—such as *abnormal patterns of brain activity, atypical brain development*, or irreversible structural brain changes—remain speculative and should be regarded as thought experiments rather than evidence-based concerns. Even the comprehensive SCHEER report did not identify evidence supporting these speculative risks and did not report adverse effects beyond those already established in the existing safety literature, although it acknowledges that the potential risks associated with frequent long-term use remain uncertain.

## V. RISK CLASSIFICATION LOGIC

Regulation (EU) 2022/2347, Recital 7, justifies the classification to Class III by referring to the possibility that induced neural modifications may result in long-lasting and potentially irreversible effects. However, the established safety literature—most recently corroborated by the SCHEER opinion—does not provide evidence for such outcomes when devices are used within well-defined, evidence-based safety limits.^45^ Consequently, the occurrence of long-lasting or irreversible effects can, at most, be considered plausible under conditions that deviate from these limits, such as frequent, unsupervised, or otherwise uncontrolled use, including misuse. Under such conditions, these effects are not intrinsic device properties but arise from deviations from controlled application.

Within the MDR, such risk considerations are reflected in the general classification rules for active devices. According to Rule 9 of Annex VIII, active therapeutic devices intended to administer or exchange energy are classified as Class IIa, unless they administer energy in a potentially hazardous way, in which case they are classified as Class IIb. Consequently, potential risks associated with frequent long-term use can be subsumed under the concept of ‘potentially hazardous energy delivery’ within the meaning of Rule 9, resulting in classification as Class IIb when applying the MDR classification logic.

This interpretation is consistent with the treatment of comparable energy-based technologies under the MDR. The MDCG 2021–24 guidance lists magnetic resonance equipment (MRI) among active diagnostic devices classified as Class IIa. Notably, MRI systems operate by generating strong static and time-varying magnetic fields, which inherently entail significant risks if not properly controlled.^46^ In addition, potential biological effects, such as DNA damage, remain under investigation, and, analogous to NIBS technologies, uncertainties persist regarding possible long-term effects of repeated exposure.^47^

These risks are not attributed to intrinsic device failure modes but are managed through controlled conditions of use, including screening procedures, standardized protocols, and trained operation. Accordingly, MRI safety is effectively ensured through well-established control measures, including strict access regulation, standardized operating procedures, and user training. Within the MDR framework, this results in a classification as Class IIa, reflecting that even devices with potentially severe adverse outcomes may remain in lower risk classes where risks are predictable, controllable, and primarily use-dependent, rather than inherent and unavoidable.

Accordingly, the regulatory framework treats these risks as controllable through design and use-related safeguards rather than as inherent device hazards. Commission Implementing Regulation (EU) 2022/2346 operationalizes this approach by requiring detailed risk control measures targeted at foreseeable misuse and unsupervised application for NIBS devices. Annex VII, Section 4.3 specifies measures such as parameter restrictions, automatic deactivation, and alarm systems to prevent exceedance of critical thresholds.

However, this regulatory logic is not consistently applied. While Regulation (EU) 2022/2346 assumes that risks can be effectively controlled through technical and procedural safeguards, Regulation (EU) 2022/2347 justifies classification as Class III based on the same risk considerations, citing the potential for long-lasting or irreversible effects arising from neuromodulation. This creates a structural inconsistency within the regulatory framework: risks that are explicitly defined as controllable in one instrument [(EU) 2022/2346] are simultaneously treated as justification for the highest risk classification in another [(EU) 2022/2347]. Such a divergence suggests that the classification decision is not fully aligned with the underlying risk management logic of the MDR, which typically reserves Class III for risks that are inherent, unavoidable, and not adequately mitigable through design or controlled use.

By contrast, Class III classification under the MDR is generally reserved for devices with an inherent high-risk profile, particularly where the device controls or directly influences the performance of active implantable devices, or where failure may lead to immediate and severe harm. NIBS devices do not exhibit such characteristics, as they neither involve invasive interaction with the body nor control life-sustaining or implantable systems.

These observations suggest a divergence between the general risk classification logic of the MDR and its application to non-medical NIBS devices. While the reclassification may be consistent with the limited evidence base used to support it, a different perspective is reflected in Regulation (EU) 2022/2346, which appears to base risk management requirements mostly on widely recognized guidelines (eg Rossi et al., 2021). This approach is also consistent with the broader empirical literature, which characterizes the safety profile of NIBS as dependent on application parameters and conditions of use. In this context, the classification raises questions regarding the alignment between the evidence considered in the reclassification and the overall regulatory approach to risk.

## VI. RISK MANAGEMENT OF ANNEX XVI DEVICES

For devices without an intended medical purpose, establishing a benefit-to-risk ratio is inherently problematic, as these products are not designed to deliver a clinical benefit that could justify exposure to health risks. Consequently, Annex XVI devices are subjected to a stricter standard—they must not present any risk at all, or only a maximum acceptable risk that is consistent with a high level of protection for human health and safety, as stipulated in Annex I, Section 9 of Regulation (EU) 2017/745. This risk threshold is further elaborated in Article 3.3 of Annex I to Regulation (EU) 2022/2346, which limits acceptable residual risks to those that are transient and do not require medical or surgical intervention to prevent life-threatening illness or permanent impairment. However, this definition is problematic in itself. As discussed by Bublitz and Ligthart, the regulation appears to adopt an absolute threshold approach, which would render any non-transient adverse effect, no matter how rare, automatically unacceptable.^48^ Paradoxically, Article 3.3 of Annex I, mentions surgical intervention as an example in which risks must be eliminated or reduced as far as possible, despite the fact that surgery inherently carries the residual risks of a sepsis-induced death. Surgery, chosen as an example here, already exceeds the threshold of maximum acceptable risk defined by the very same article. This illustrates the fundamental problem with an absolute risk threshold: it does not account for the probability of harm. As a result, many devices, including those for TMS, which pose extremely low-probability risks will still technically exceed the rigid threshold, making their approval questionable despite a favorable overall safety profile.

In principle, the acceptable risk criterion of Article 3.3 of Annex I would make it impossible to certify any non-medical device that involves a surgical procedure (such as devices defined under Annex XVI, points 2 and 4, MDR) or any other devices that carry residual risks exceeding the defined threshold. However, a notable exception is included within the same article. It permits manufacturers to justify the acceptability of such risks when one or more of the specified conditions are not fulfilled. This vague and open-ended clause introduces significant uncertainty, as it relies on case-by-case judgment and may be influenced by the subjective interpretation of the responsible authority. In consequence, this subjectivity creates a lack of legal predictability and increases the risk of inconsistent regulatory decisions.

By allowing only transient residual risks, the regulation implicitly treats plausible yet extremely rare events as categorically unacceptable.^49^ As a result, many devices—despite having a favorable overall safety profile—fall within this strict definition, creating unintended inconsistencies within EU law. A probability-weighted assessment of risk, in line with established principles of medical device regulation, would provide a more realistic and coherent framework.

The SCHEER opinion provides a comprehensive and structured synthesis of the available literature and contributes to clarifying the current state of evidence.^50^ However, it does not establish a basis for a probability-weighted assessment of risk, as it does not provide quantitative estimates of the likelihood of adverse events nor a systematic integration of severity and probability under defined conditions of use. Risk considerations are primarily framed qualitatively and in relation to contextual factors such as application parameters and user conditions. Consequently, while the opinion strengthens the qualitative characterization of potential risks, it does not, in its current form, enable a fully probability-weighted evaluation of overall risk.

## VII. SIGNALING POWER OF LAW

Law does not only regulate specific subject matters; it also has an expressive function that shapes social norms and beliefs.^51^ The reclassification of non-medical NIBS devices into the highest risk class therefore influences how the technology is perceived, potentially extending its impact to medical NIBS applications as well. The mere fact that such claims are codified in law and issued by a legitimate authority (EU) lends credibility, so that even unsubstantiated side effects listed in the regulation may be accepted as real. This dynamic can be explained by the *Condorcet Jury Theorem*,^52^ which suggests that legislative decisions are often perceived as more informed than those of individual actors. In addition, the perceived legitimacy of the legal authority itself can foster uncritical acceptance of what the law states.^53^ Taken together, these mechanisms render the inclusion of unsubstantiated side effects in a legal text especially concerning, since their impact extends well beyond the regulation’s intended scope.

### VII.A. Practical Implications

Consequences of the signaling power and the perceived legitimacy of legal texts are not merely theoretical but have already manifested in practice. For instance, the classification of non-medical purpose NIBS devices as medical devices under Annex XVI led some stakeholders, including ethics committees, to assume that all related research studies must be considered ‘other’ clinical investigations.^54^ As we have previously clarified,^55^ and as explicitly confirmed in MDCG 2021–6 Rev. 1 (see, eg Question 19), this interpretation is incorrect. Nevertheless, in one case an institutional ethics committee suspended all votes on research involving NIBS devices—including those intended for medical purposes—when it encountered the newly listed potential side effects in Regulation (EU) 2022/2347. This decision was taken irrespective of empirical evidence and despite the fact that the scientific literature cited in support of the regulation is demonstrably inadequate to justify such claims. This reaction illustrates in concrete terms the signaling power of law described above: the codification of potential risks in legislation was sufficient to shape institutional judgment and practice, even in the absence of supporting evidence.

Beyond this immediate institutional response, such signaling effects carry wider and longer-term implications for the research ecosystem. In particular, they risk creating a chilling effect that not only obstructs individual research projects but also reshapes career choices, research priorities, and the geographic distribution of scientific expertise. Early academic careers are typically structured around short-term contracts of two to three years. Given that Regulation (EU) 2022/2347 has now been in force for nearly three years, an entire generation of early-career researchers may already have made strategic decisions to avoid NIBS research altogether or to redirect their careers toward fields perceived as less encumbered by regulatory uncertainty. This not only reduces the pool of expertise in Europe but also risks contributing to a ‘brain drain’, as young scientists may choose to pursue opportunities in regions where research on NIBS devices is less stigmatized or obstructed. Left unaddressed, the long-term consequence could be a structural weakening of European research capacity in neuroscience and neurotechnology, which would further translate into a reduction of innovation and availability of new methods or technologies.

## VIII. DIVERSITY OF DEVICES

The groups of non-medical brain stimulation devices covered by the Regulations 2022/2346 and 2022/2347 differ considerably in their technical complexity, risk profile, costs, and potential markets. Transcranial electrical stimulation (tES) devices can be constructed as do-it-yourself systems for as little as 50€ (DIY still unregulated), with commercially available high-end variants costing around 8000€. By contrast, TMS systems require high voltages, sophisticated components, and complex engineering, and are typically sold at prices between 80,000€ and 120,000€. While the Regulations are designed with consumer-oriented neuroenhancement products in mind, where a plausible lay market exists for relatively affordable tES devices, it is highly implausible that such a market exists for current TMS devices due to their technical complexity and price points. Realistically, non-medical TMS systems would likely only be procured for research institutions, where they are operated by highly educated research users with advanced training and subject to oversight from ethics committees. For other Annex XVI device categories, such as intense pulsed light systems or adipose tissue reduction equipment, the regulations explicitly differentiate between professional and lay use. Extending a similar distinction to neurostimulation devices would therefore be consistent with regulatory practice and more proportionate to the actual risk and market realities.

### VIII.A. User Groups

A proportionate regulatory framework could explicitly differentiate between distinct user groups. Consumer lay users represent the group most in need of stringent safeguards, as they typically lack the expertise required for safe use and are most vulnerable to misuse. Professional users in commercial contexts, potential neuroenhancement, or wellness centers, occupy an intermediate position: although operating outside the medical system, their activities take place in structured environments where training requirements and facility-level obligations can be imposed. Finally, research users—highly qualified scientists and clinicians within academic or clinical institutions—work under established scientific protocols and are subject to mandatory ethical oversight by institutional review boards. Recognizing these distinctions in law would allow for graduated regulatory requirements that align with actual competence levels and oversight mechanisms, rather than applying an undifferentiated Class III designation across all contexts.

Since NIBS devices with a medical purpose remain to be classified in risk class IIa or IIb, despite the same risk profile, instead of imposing non-medical NIBS devices to the highest risk class, a more proportionate solution would be to introduce this tripartite categorization—consumer, professional, and research devices. The current blanket classification as risk Class III appears calibrated to the most critical user group and use scenario, but it lacks the specificity to account for contexts where risks can be effectively controlled by normative requirements such as the common specifications that can be tuned to specific user groups or use scenarios.

## IX. CONCLUSIONAL REMARKS

The results of this analysis indicate limitations in the application of evidence-based principles within the regulatory process. Although the European Commission emphasizes that legislative decisions should rely on scientific evidence, the reclassification of non-medical NIBS devices appears to have been based on literature that is unsuitable for evaluating the safety or risks of NIBS devices. The cited sources to support the reclassification do not investigate the adverse effects or safety of NIBS devices and therefore cannot serve as an adequate evidentiary basis for such a decision. At the same time, this narrow selection disregards a substantial body of empirical research—including hundreds of high-quality clinical trials and meta-analyses—with a broad consensus on side effects. The explicit reliance on five non-empirical sources, despite the availability of extensive clinical data, suggests that existing internal review and quality assurance procedures may not have been sufficient to identify this imbalance before adoption. In this context, the procedural significance of the endorsement by the MDCG warrants further consideration, particularly regarding how scientific evidence is selected, weighted, and integrated into the lawmaking process. However, the subsequent involvement of the European Ombudsman and the decision to mandate an independent reassessment by the SCHEER committee provide reason for optimism, suggesting that secondary corrective mechanisms within the EU’s legislative framework do function when concerns are raised. The SCHEER opinion, with its structured and comprehensive review of the available literature, is expected to establish a more appropriate and representative evidence base for assessing the safety profile of NIBS.^56^ At the same time, it must be acknowledged that Regulation (EU) 2022/2347 has already been in force for more than two years, and a timely regulatory correction remains uncertain. Consequently, any evidence-based reassessment is likely to occur with considerable delay, prolonging the current misalignment between regulatory classification and the underlying scientific evidence.

Taken together, these findings underscore the need for a revised regulatory framework that ensures safety without erecting disproportionate barriers. Possible solutions include:

**(i) User competence-centered regulation instead of highest risk class**, allowing regulatory requirements to be adapted to different user groups and use environments. Such differentiation would reflect a core principle of the MDR, namely that risks should be managed in relation to foreseeable use and user characteristics. This improves proportionality and helps to avoid overregulation in contexts where risks can be effectively controlled through training, supervision, and procedural safeguards.

**(ii) Adopting risk-based rather than absolute thresholds**, ensuring that regulatory decisions account not only for the severity but also for the probability of harm. This would align the framework with established risk management principles (eg ISO 14971) and the broader logic of the MDR, which is based on a balance between benefit, risk, and likelihood, rather than the exclusion of technologies on the basis of hypothetical or extremely low-probability adverse events.

**(iii) Grounding regulatory decisions in robust empirical evidence**, ensuring that classification and safety requirements are derived from comprehensive and methodologically appropriate data. This reflects the principle of evidence-based policymaking embedded in EU governance and supports the scientific validity, transparency, and legitimacy of regulatory decisions, particularly where precautionary measures are invoked.

**(iv) Ensuring meaningful consultation with domain experts**, strengthening procedural quality and consistency in the regulatory process. Early and structured integration of field-specific expertise, as exemplified by the subsequent involvement of the SCHEER committee, ensures accurate interpretation of scientific evidence, and helps to reduce the risk of regulatory misalignment, and to enhance legal certainty by ensuring that regulatory assumptions correspond to established scientific and clinical practice.

Such adjustments would not only uphold the commitment to evidence-based lawmaking but also strike a more appropriate balance between safety, innovation, and the advancement of scientific knowledge.

## Supplementary Material

Supplemental_data_lsag021

## Footnotes

1 Amitabh Bhattacharya et al., *An Overview of Noninvasive Brain Stimulation: Basic Principles and Clinical Applications*, 49 Can. J. Neurol. Sci. 479 (2022).

2 Tad T. Brunyé, *Non-Invasive Brain Stimulation Effects on the Perceptual and Cognitive Processes Underlying Decision-Making: A Mini Review*, 5 J. Cogn. Enhanc. 233 (2021).

3 Fatemeh Ehsani et al., *The Effects of Transcranial Direct Current Stimulation Montages on Motor Learning across Various Brain Regions: A Systematic Review and Network Meta-Analysis*, 569 Neuroscience 32 (2025).

4 Adelaide Austin et al., *Prefrontal Electrical Stimulation in Non-Depressed Reduces Levels of Reported Negative Affects from Daily Stressors*, 7 Front. Psychol. (2016), http://journal.frontiersin.org/Article/10.3389/fpsyg.2016.00315/abstract.

5 Roneil G. Malkani & Phyllis C. Zee, *Brain Stimulation for Improving Sleep and Memory*, 15 Sleep  Med. Clin. 101 (2020).

6 Claudio Lucchiari, Paola Maria Sala & Maria Elide Vanutelli, *Promoting Creativity Through Transcranial Direct Current Stimulation (tDCS). A Critical Review*, 12 Front. Behav. Neurosci. 167 (2018).

7 Kathrin Guerlich et al., *European Expert Recommendations on Clinical Investigation and Evaluation of High-risk Medical Devices for Children*, 112 Acta  Paediatrica 2440 (2023); Tom Melvin et al., *Orphan Medical Devices and Pediatric Cardiology – What Interventionists in Europe Need to Know, and What Needs to Be Done*, 44 Pediatr. Cardiol. 271 (2023).

8 Andrea Antal et al., *The Consequences of the New European Reclassification of Non-Invasive Brain Stimulation Devices and the Medical Device Regulations Pose an Existential Threat to Research and Treatment: An Invited Opinion Paper*, 163 Clinical  Neurophysiology 280 (2024); Chris Baeken et al., *European Reclassification of Non-Invasive Brain Stimulation as Class III Medical Devices: A Call to Action*, 16 Brain  Stimulation 564 (2023); Walter Paulus, *European Medical Device Regulation: Perfection Is Achieved, Not When There Is Nothing More to Add, but When There Is Nothing Left to Take Away*, 163 Clin. Neurophysiol 265 (2024).

9 Baeken et al., *supra* note 8.

10
*Better Regulation - European Commission*, https://commission.europa.eu/law/law-making-process/better-regulation_en (accessed Oct. 23, 2025).

11 Balder Onarheim, *EU Regulators Grossly Misrepresent Non-Invasive Brain Stimulation*, neuromodec J (2023), https://neuromodec.org/2023/01/eu-regulators-grossly-misrepresent-non-invasive-brain-stimulation/.

12
*SCHEER - Opinions - Public Health - European Commission*, https://health.ec.europa.eu/scientific-committees/scientific-committee-health-environmental-and-emerging-risks-scheer/scheer-opinions_en (accessed Mar. 25, 2026).

13 Bhattacharya et al., *supra* note 1; Anli Liu et al., *Immediate Neurophysiological Effects of Transcranial Electrical Stimulation*, 9 Nat. Commun. 5092 (2018).

14 Paolo M. Rossini & Simone Rossi, *Transcranial Magnetic Stimulation: Diagnostic, Therapeutic, and Research Potential*, 68 Neurology 484 (2007).

15 Tad T. Brunyé et al., *Cranial Electrotherapy Stimulation (CES) Does Not Reliably Influence Emotional, Physiological, Biochemical, or Behavioral Responses to Acute Stress*, 6 J. Cogn. Enhanc. 417 (2022).

16 Hannah Maslen et al., *The Regulation of Cognitive Enhancement Devices: Extending the Medical Model*, 1 J. Law  Biosci. 68 (2014); Hannah  Maslen et al., Mind  Machines: The  Regulation of  Cognitive  Enhancement  Devices, Oxford Martin School (2014).

17 Novel Neurotechnologies: Intervening in the Brain (2013).

18 Katia Monte-Silva et al., *Induction of Late LTP-Like Plasticity in the Human Motor Cortex by Repeated Non-Invasive Brain Stimulation*, 6 Brain  Stimulation 424 (2013).

19 Katy Wagner et al., *Would You Be Willing to Zap Your Child’s Brain? Public Perspectives on Parental Responsibilities and the Ethics of Enhancing Children with Transcranial Direct Current Stimulation*, 9 AJOB Empirical  Bioethics 29 (2018).

20 Andrea Antal et al., *Note of Concern Regarding the Sources of Scientific Evidence Used to Justify the Reclassification of Non-Invasive Brain Stimulation (NIBS) Devices without an Intended Medical Purpose into Class III*, 18 Brain  Stimulation 103 (2025).

21
*Id.*

22 Maslen et al., *supra* note 16.

23 Maslen et al., *supra* note 16.

24 Megan Spencer-Smith & Vicki Anderson, *Healthy and Abnormal Development of the Prefrontal Cortex*, 12 Dev. Neurorehabil. 279 (2009).

25 Roi Cohen Kadosh et al., *The Neuroethics of Non-Invasive Brain Stimulation*, 22 Curr. Biol. R108 (2012).

26 Roi Cohen Kadosh et al., *Modulating Neuronal Activity Produces Specific and Long-Lasting Changes in Numerical Competence*, 20 Curr. Biol. 2016 (2010).

27 Novel Neurotechnologies: Intervening in the Brain, *supra* note 17.

28
*Id.* at vii.

29 Simone Rossi et al., *Safety, Ethical Considerations, and Application Guidelines for the Use of Transcranial Magnetic Stimulation in Clinical Practice and Research*, 120 Clin. Neurophysiol. 2008 (2009).

30 Novel Neurotechnologies: Intervening in the Brain, *supra* note 15, at 22.

31 Monte-Silva et al., *supra* note 18.

32 Wagner et al., *supra* note 19.

33
*Id.*

34 Antal et al., *supra* note 20.

35 A.T. Barker, R. Jalinous & I.L. Freeston, *Non-Invasive Magnetic Stimulation of Human Motor Cortex*, 325 The  Lancet 1106 (1985).

36
Practical  Guide to  Transcranial  Direct  Current  Stimulation: Principles, Procedures and  Applications (Helena Knotkova et al. eds., 2019), https://link.springer.com/10.1007/978-3-319-95948-1.

37 M. A. Nitsche & W. Paulus, *Excitability Changes Induced in the Human Motor Cortex by Weak Transcranial Direct Current Stimulation*, 527 J. Physiol. 633 (2000).

38 Wanalee Klomjai, Rose Katz & Alexandra Lackmy-Vallée, *Basic Principles of Transcranial Magnetic Stimulation (TMS) and Repetitive TMS (rTMS)*, 58 Ann. Phys. Rehabil. Med. 208 (2015); Liu et al., *supra* note 13.

39 Matthew J. Burke, Peter J. Fried & Alvaro Pascual-Leone, *Transcranial Magnetic Stimulation: Neurophysiological and Clinical Applications*, 163 Handb. Clin. Neurol. 73 (2019), https://linkinghub.elsevier.com/retrieve/pii/B9780128042816000057.

40 Jean-Pascal Lefaucheur et al., *Evidence-Based Guidelines on the Therapeutic Use of Transcranial Direct Current Stimulation (tDCS)*, 128 Clin. Neurophysiol. 56 (2017); Simone Rossi et al., *Safety and Recommendations for TMS Use in Healthy Subjects and Patient Populations, with Updates on Training, Ethical and Regulatory Issues: Expert Guidelines*, 132 Clin. Neurophysiol. 269 (2021).

41 SCHEER - Opinions - Public Health - European Commission, *supra* note 12.

42
*Id.*

43
*Id.*

44 Antal et al., *supra* note 20; Baeken et al., *supra* note 8.

45 SCHEER - Opinions - Public Health - European Commission, *supra* note 12.

46 Love Engström Nordin et al., *ESR Essentials: Basic Physics of MR Safety—Practice Recommendations by the European Society for Magnetic Resonance in Medicine and Biology*, 35 Eur. Radiol. 572 (2024); Jana G. Delfino et al., *MRI-related FDA Adverse Event Reports: A 10-yr Review*, 46 Med. Phys. 5562 (2019).

47 M.A. Hill, P. O’Neill & W.G. McKenna, *Comments on Potential Health Effects of MRI-Induced DNA Lesions: Quality Is More Important to Consider than Quantity*, 17 Eur. Heart J. Cardiovasc. Imaging 1230 (2016).

48 Christoph Bublitz & Sjors Ligthart, *The New Regulation of Non-Medical Neurotechnologies in the European Union: Overview and Reflection*, 11 J. Law  Biosci. lsae021 (2024).

49
*Id.*

50 SCHEER - Opinions - Public Health - European Commission, *supra* note 12.

51 Cass R. Sunstein, *On the Expressive Function of Law*, 144 U. Pa. L. Rev. 2021 (1996).

52 D. Dharmapala, *The Condorcet Jury Theorem and the Expressive Function of Law: A Theory of Informative Law*, 5 American  Law and  Economics  Association 1 (2003).

53
Tom R. Tyler, Why People Obey the Law (2006).

54 Antal et al., *supra* note 8.

55 Roman Rethwilm et al., *Commentary: Do All Studies Using Medical Devices Fall under the European Medical Device Regulation?*, 167 Clin. Neurophysiol 239 (2024).

56 SCHEER - Opinions - Public Health - European Commission, *supra* note 12.

