# Supplementary material for: From speculation to regulation: an evidence-based analysis of the EU reclassification of non-invasive brain stimulation devices
Source: J Law Biosci. 2026 Aug 26;13(2):lsag021. doi: 10.1093/jlb/lsag021 (PMC13518052; doi:10.1093/jlb/lsag021)
Supplement: Supplementary file 1 [file supplemental_data_lsag021.pdf]

PRISMA 2020 flow diagram for new systematic reviews which included searches of databases and registers only

Supplemental data

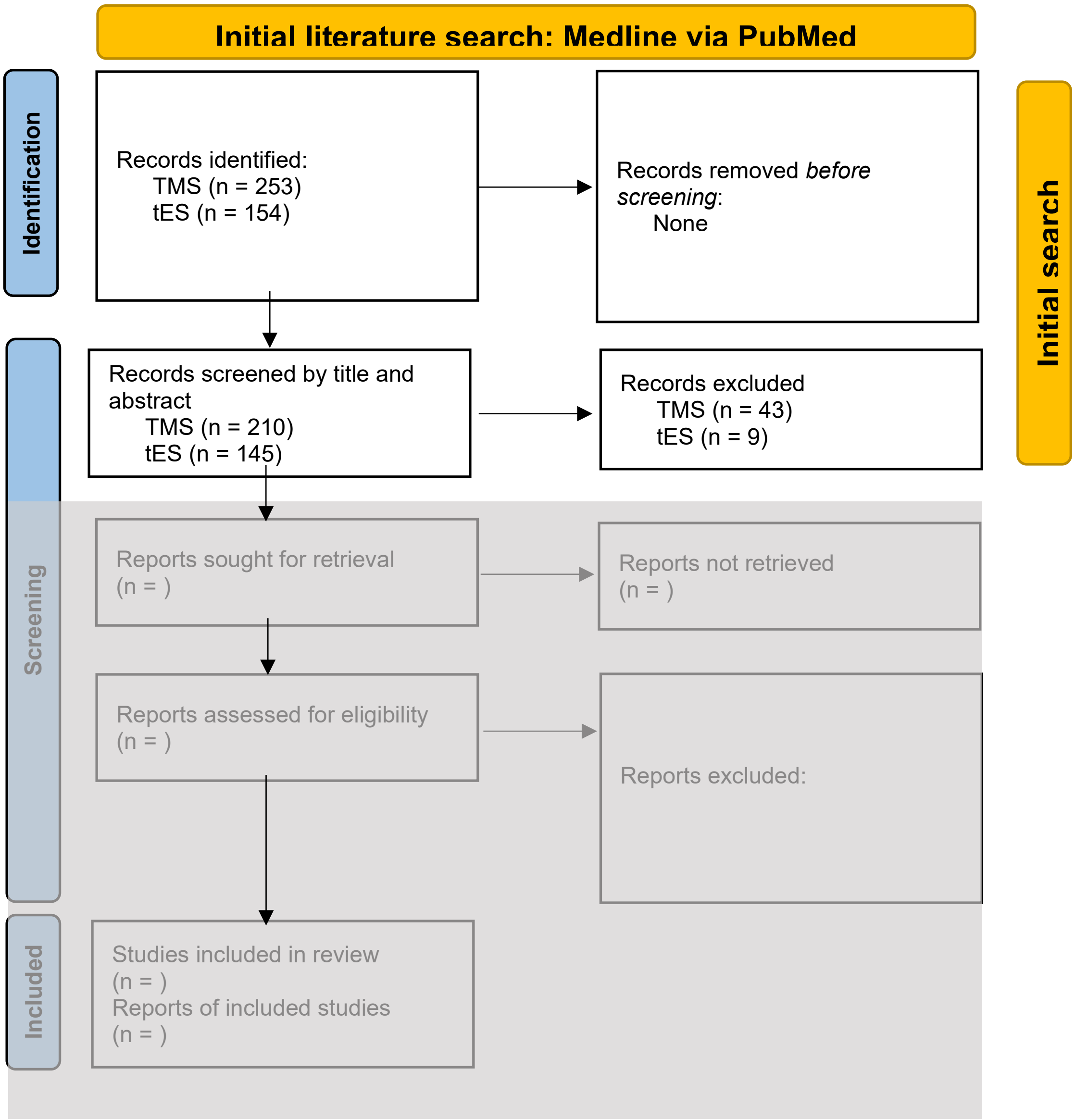

Search query TMS:

("transcranial magnetic stimulation"[Title/Abstract] OR TMS[Title/Abstract]) AND safety[Title/Abstract] AND ((clinicaltrial[Filter] OR controlledclinicaltrial[Filter] OR randomizedcontrolledtrial[Filter]) AND (humans[Filter]))

Search query tES:

("transcranial direct current stimulation"[Title/Abstract] OR tDCS[Title/Abstract] OR "transcranial alternating current stimulation"[Title/Abstract] OR tACS OR "transcranial random noise stimulation"[Title/Abstract] OR tRNS[Title/Abstract]) AND safety[Title/Abstract] AND ((clinicaltrial[Filter] OR controlledclinicaltrial[Filter] OR randomizedcontrolledtrial[Filter]) AND (humans[Filter]))

Date of search:

17th October 2025

Source: Page MJ, et al. BMJ 2021;372:n71. doi: 10.1136/bmj.n71.

This work is licensed under CC BY 4.0. To view a copy of this license, visit https://creativecommons.org/licenses/by/4.0/
